# Electronically Controlled Time-Domain Integral Average Depolarizer Based on a Barium Titanate (BTO) Metasurface

**DOI:** 10.3390/nano12071228

**Published:** 2022-04-06

**Authors:** Kaiqian Jie, Hui Huang, Shuai Qin, Jianping Guo, Hongzhan Liu, Hongyun Meng, Faqiang Wang, Xiangbo Yang, Zhongchao Wei

**Affiliations:** Guangdong Provincial Key Laboratory of Nanophotonic Functional Materials and Devices, School of Information and Optoelectronic Science and Engineering, South China Normal University, Guangzhou 510006, China; kqjie@m.scnu.edu.cn (K.J.); huihuang@m.scnu.edu.cn (H.H.); shuaiqin@m.scnu.edu.cn (S.Q.); guojpgz@163.com (J.G.); lhzscnu@163.com (H.L.); hymeng@scnu.edu.cn (H.M.); fqwang@scnu.edu.cn (F.W.); xbyang@scnu.edu.cn (X.Y.)

**Keywords:** depolarizer, time-domain integral average, metasurface, barium titanate (BTO)

## Abstract

A depolarizer, a kind of optical element that converts polarized light to unpolarized light, has been found massive applications in classical optics. However, depolarizers based on metasurface which can be applied in integrated optics have rarely been proposed. In this paper, an electronically controlled metasurface depolarizer is demonstrated based on the time-domain integral average method and nano-material barium titanate. It obtains emergent light with a degree of polarization reduced to 2.5% when hit by linearly polarized light at 633 nm, and has a transmission efficiency greater than 72%. This depolarizing metasurface can be designed on-demand, immunizing the degree of the emergent light from its size, and has the simple electronic control with high-speed response.

## 1. Introduction

Polarization is one of the fundamental properties of light, so the control of it has an irreplaceable interest in the development of optics. However, polarization is useless or even harmful to the accuracy of instruments for certain optical systems [1]. For example, in some medical laser systems, the skin’s absorption of energy varies according to the polarization state of laser beams, so it is better to use unpolarized light in some medical treatments to avoid damage. Additionally, in long-haul fiber-optical communication systems with polarization-sensitive devices (e.g., connectors, switches, couplers, or isolators), the randomly state of polarization gives rise to intensity noise [2,3,4,5].

Depolarizers are designed to lower the degree of polarization (DoP) of polarized light [1]. A classic type of depolarizer used in a real beam-polarization sensing system is the Lyot depolarizer, proposed by Lyot in 1928 [6]. This method achieves light with a low DoP by scrambling the polarization of incident light at different wavelengths, which relied on the dispersion of the phase retardation of crystals. The disadvantage of this kind of depolarizers is that they need to work on broadband resources to achieve as low a DoP as possible and cannot meet the depolarization requirements of monochromatic lasers [7,8,9,10,11]. With rapid development towards working with monochromatic laser beams of optical elements, depolarizers with advantages of high-efficiency and integration had been proposed in succession. For example, Billings implemented a depolarizer working with monochromatic beam in 1951, which is a dynamically regulated depolarizer composed of two waveplates that change with voltage [12]. However, the depolarizer is bulky and requires extremely high voltage, which limits its applications. Subsequently, the development of liquid crystal (LC) materials in the 21st century gave birth to many LC-based depolarizers [13,14,15,16]. These devices are very large in pixel size, calling for beams of the incident light with several hundred micrometers in diameter. Moreover, the devices are made of bulk LC, making them difficult to further develop in integrated optics. It is worth noting that, in recent years, the LC has been perfectly combined with a metasurface to achieve many tunable two-dimensional devices with great potential for application [17,18,19,20]. Predecessors have tried to implement depolarizers in the field of surface plasmons, but the inherent properties of the metal material itself limit the efficiency of the devices [21,22,23].

A metasurface, with its unprecedented light modify capabilities, provides an unusual method for polarization control and has made significant progress in the development of it, such as polarization imaging [24,25,26] and optical holography [27,28,29,30,31,32]. In 2020, Ting proposed an all-dielectric metasurface depolarizer with a spatial-domain average method [33]. The device is composed of nanopillars acting as half-waveplates with a random fast-axis, which can obtain emergent light with random polarization directions when hit by linear polarized light. The device can depolarize beams as small as 10 μm in diameter, and it is about two orders of magnitude smaller than commercial liquid crystal depolarizers. However, since this kind of depolarizer is implemented based on the spatial average method, the size of the element needs to be large enough to obtain the lower DoP.

**Figure 1:**
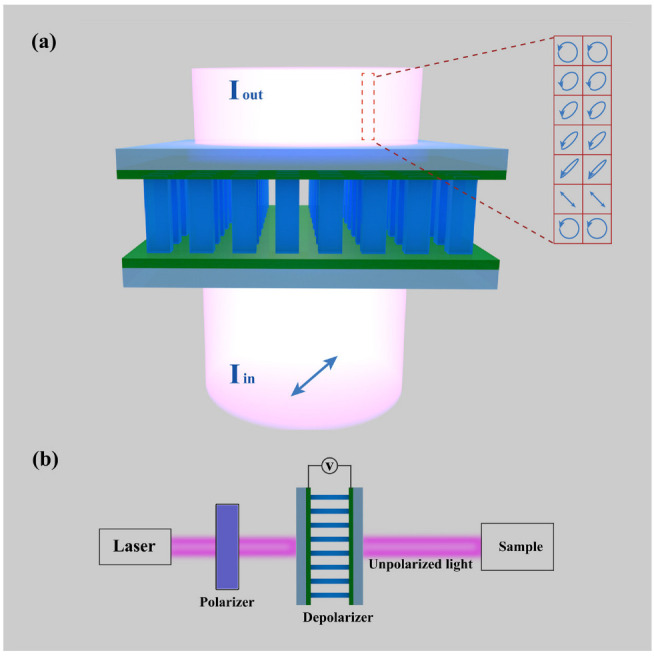
The schematic of the metasurface depolarizer. (**a**) The incident light is linearly polarized light with polarization direction angle α=π4, and after passing through the metasurface depolarizer, the emergent light obtained a low DoP with random states of polarization. (**b**) Example of general use of depolarizers.

This paper demonstrates a time-domain integral average depolarizer based on a barium titanate (BTO) metasurface, which achieves the emergent light with low DoP by applying different voltages. The time-domain integral average method is derived from the method of using temporally varying retarders to depolarize [12], which lifts the size restrictions of the depolarizer device, allowing the size of it to be designed according to the beam size and target needs. The use of BTO, a kind of electronically controlled phase change material, provides a more accurate and convenient control method for the depolarizer, which greatly improves its response speed. The metasurface depolarizer we proposed is shown in Figure 1. It consists of an array of BTO nanopillars, designed to act as nanoscale waveplates whose function can change with the voltage. When the applied voltage changes continuously, different polarization states of the output light can be obtained instantly, so as to realize the depolarization in time-domain integral average. The element uses two layers of indium tin oxide (ITO) films for uniform and undifferentiated voltage applied to it from 0 V to 30 V. We conducted simulation verification and found that the DoP of the emergent light can be reduced to 2.5% with the minimum efficiency higher than 72%. This kind of metasurface depolarizer possesses additional merits, such as high performance, easy fabrication, multi-function and tunable compact configuration, allowing them to meet the requirements of modern integrated optics.

## 2. Materials and Methods

**Figure 2:**
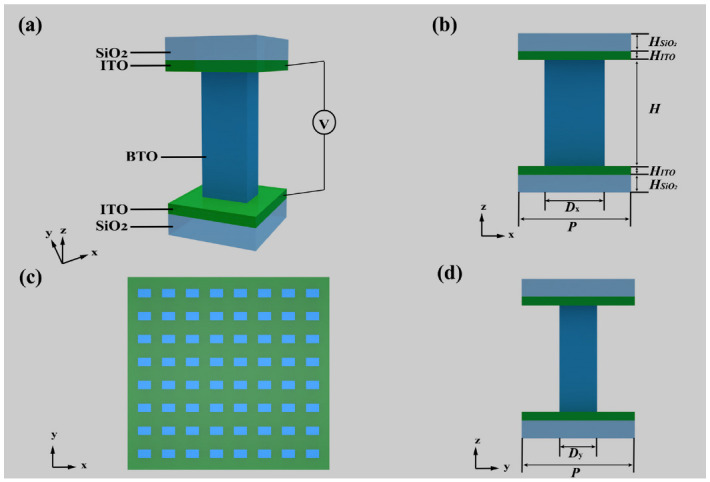
Schematic diagram of the meta-atom and its placement, in which (**a**) the perspective view, (**b**) front view and (**d**) left side view and (**c**) the placement are shown. The meta-atom of the depolarizer is composed of a BTO nanopillar, and two layers of ITO and SiO_2_ substrates. The lattice constant of unit *P* is 350 nm; the heights of the SiO_2_, ITO and BTO materials, respectively, are *H*_SiO__2_ = 200 nm, *H*_ITO_ = 50 nm and *H* = 590 nm; and the length and width of the BTO nanopillars, respectively, are *D*x = 242 nm and *D*y = 100 nm. The voltage is applied to the BTO through two layers of ITO electrodes. (**c**) The meta-atom is placed with a rotation angle θ=0 to make their simpler to produce.

As shown in Figure 2, the meta-atom of the depolarizer is composed of a BTO nanopillar, two layers of ITO and SiO_2_ substrates. The voltage was applied on the ITO electrodes to change the effective refractive index of the BTO.

The metasurface is designed to be a quarter-waveplate when no voltage is applied (*V* = 0) and transferred to a half-waveplate when the applied voltage becomes 30 V. When the applied voltage changes from 0 V to 30 V uniformly and rapidly, the metasurface acts as numbers of waveplates with different phase shift, which acts as a depolarizer.

### 2.1. The Electronically Adjustable Waveplates

The tunable property with electronically driven control of method is quiet a hotspot in metasurface. BTO is a mature electronically controlled phase change material due to its electro-optical (EO) effect, named Pockels effect. The BTO material can still own a strong Pockels effect and an ultra-fast (sub-ps) modulation speed even in nanoscale, maintaining the stability of chemical and thermal properties at the same time [34]. Additionally, BTO has a low absorption in visible region, which can meet the need of working with 633 nm light of the He-Ne laser in this paper. The equation for the relationship between the ordinary refractive index of BTO and the applied voltage is shown below [35],

(1) $$n=n_{0}+\frac{1}{2}n_{0}^{3}r_{51}V/H$$

where $n_{0}=2.4$ is the real part of the refractive index of BTO in the state of nature, the EO coefficient $r_{51}$ is 923 pm/V in nanoscale according to the [34]. *V* is the voltage applied externally to BTO nanopillars through ITO layers, and *H* is the height of BTO nanopillar. From the formula, the refractive index of the BTO with a fixed structure can be calculated with the applied voltage *V*.

### 2.2. Implementation of Depolarizers

A linearly polarized light was incident perpendicularly on the metasurface, and its polarization direction is at an angle α to the x axis. At the same time, we use the ITO layers to apply a continuously and rapidly changing voltage to the metasurface. Upon transmission, the incident light can be transformed into an emergent light with random polarization directions and thereby the element significantly reduces the DoP of light.

To further quantify the performance of the depolarizers, the DoP was defined as [1],

(2) $$\mathrm{DoP}=\frac{\sqrt{S_{1}{}^{2}+S_{2}{}^{2}+S_{3}{}^{2}}}{S_{0}}\times 100\%$$

where $S_{0}$,$S_{1}$,$S_{2}$, and $S_{3}$ are the four fundamental parameters of the Stokes vector S. To facilitate the derivation of the process of a beam passing through a metasurface, we use the Stokes and Muller calculus. The $S_{\mathrm{in}}={\left(\begin{array}{cccc} S_{0}, & S_{1}, & S_{2}, & S_{3} \end{array}\right)}^{T}$ is used to represent the Stokes vector of incident light while the Sout=(S0',S1',S2',S3')T of the beam passing through the waveplates. Additionally, the Mueller matrix of the metasurface acts as a waveplate with the phase shift ϕ, and the rotation angle θ is M, a 4 × 4 real matrix [1],

(3) $$M=\left(\begin{array}{cccc} 1 & 0 & 0 & 0\\ 0 & \cos^{2}2\theta +\cos\phi \sin^{2}2\theta & (1-\cos\phi )\sin2\theta \cos2\theta & -\sin\phi \sin2\theta \\ 0 & (1-\cos\phi )\sin2\theta \cos2\theta & \sin^{2}2\theta +\cos\varphi \cos^{2}2\theta & \sin\phi \cos2\theta \\ 0 & \sin\phi \sin2\theta & -\sin\phi \cos2\theta & \cos\phi \end{array}\right)$$

In this paper, we set the nanopillars’ rotation angle θ to zero for calculation simplification and manufacturing convenience.

When a normal linear polarization light with a polarization direction angle α with respect to the x axis passes through the metasurface element, the process can be described as $S_{\mathrm{out}}={MS}_{\mathrm{in}}$, so the Stokes vector of the emergent beam $S_{\mathrm{out}}$ can be written as follows:

(4) Sout=(S0'S1'S2'S3')=(1000010000cosϕsinϕ00−sinϕcosϕ)(1cos2αsin2α0)=(1cos2αsin2αcosϕ−sin2αsinϕ)

The $S_{\mathrm{out}}$ stands for a general elliptically polarized light with a polarization direction angle α and phase shift ϕ. 

The ϕ will change as the applied voltage changes, so that it can be written as a function of ϕ(t)=2πtT, where the *t* is the time, the T is the period of changing phase shift from 0 to 2π. Hence, the polarization of emergent light can be regarded as the time average effect of polarization components, expressed as

(5) S¯out =(S¯0'S¯1'S¯2'S¯3')=(1cos2αsin2α⋅1t∫0tcosϕ(t)dt−sin2α⋅1t∫0tsinϕ(t)dt)=(1cos2αsin2α⋅sinXX−sin2α⋅(1−cosXX))

Here, X=2πtT.

Therefore, the DoP of the ${\bar{S}}_{\mathrm{out}\;}$ is

(6) $$\mathrm{DoP}=\sqrt{\cos^{2}2\alpha +\frac{4\sin^{2}\left(\frac{X}{2}\right)\sin^{2}2\alpha }{X^{2}}}$$

From the Equation (6), we can draw the image of the DoP with respect to X and α as follows.

**Figure 3:**
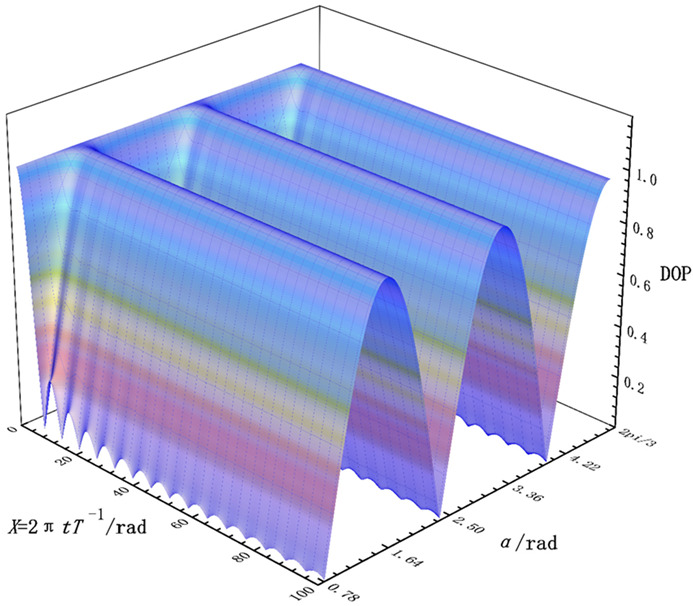
The DoP of ${\bar{S}}_{\mathrm{out}}$ when the rotation angle θ=0 and incident light is linearly polarized light with polarization direction angle α.

From Figure 3, we can know that, when the incident light is linearly polarized light, the DoP is related to the polarization direction angle α of incident light, showing a periodic oscillation relationship. Further, when $\alpha =\left(1+k\right)\frac{\pi }{4},k=0,1,2,3,\ldots ,n$, the DoP of the emergent light drops sharply as time increases. This provides theoretical support for the realization of the depolarizer. It is worth noting that, when the incident light becomes circularly polarized light, with the Stokes Matrix $S_{\mathrm{CP}}={\left(\begin{array}{cccc} 1, & 0, & 0, & \pm 1 \end{array}\right)}^{T}$, the DoP of the S¯out' is

(7) DoP'=1X2-2cosX

According to the Equation (7), DoP’ gradually tends to 0 as time increases, which proves that this method can effectively depolarize circularly polarized light, too.

## 3. Results and Discussion

When the depolarizer’s working wavelength is 633 nm, fitting with the He-Ne laser, the dimensions of the unit structure are calculated by simulation followed by a series of measurements. To obtain the phase shift between the long and short axes, we needed to choose a shape that differentiated between the long and short axes, and for ease of production, we chose a rectangle rather than an ellipse to design our unit structure. As shown in Figure 2, the meta-atom is designed to be made of five layers, and from top to bottom in sequence are the SiO_2_ substrate, the ITO transparent electrode, a BTO nanopillar, the ITO transparent electrode and the SiO_2_ substrate. To obtain an appropriate size of the BTO nanopillars with a high polarization conversion efficiency and high transmittance, we used the finite-difference time-domain (FDTD) method to simulate. Here, we chose the software FDTD solutions (Lumerical Inc., Vancouver, BC, Canada) to calculate the dimensions of a BTO nanopillar with the normal incidence of plane waves (λ=633nm) from the substrate.

We obtained the refractive index data for ITO from the website (refractiveindex.info) and the refractive index data for SiO_2_ (SiO_2_(Glass)-Palik) from the software FDTD solutions (Lumerical Inc., Vancouver, BC, Canada) materials library. A meta-atom was selected for simulation, and we applied the Periodic boundary condition in the x and y directions, respectively, to simulate the overall effect of the metasurface in a real situation. In the z direction, a perfectly matched layer (PML) was set to reduce reflection. We first fixed an identical lattice constant of *P* = 350 nm of the unit, and then nested sweeping three parameters, the height *H*, the width *D*y, and the length *D*x of BTO. Combined with data analysis, the *H*, the height of nanopillars was further determined to be 590 nm. Then, we finely swept the *D*x and *D*y to obtain the right dimensions with which the adjacent nanopillars can excite electric and magnetic Mie-type resonances resulting in high transmission and large phase shifts [36]. Finally, we obtained the size of BTO as *D*x = 242 nm and *D*y = 100 nm.

**Figure 4:**
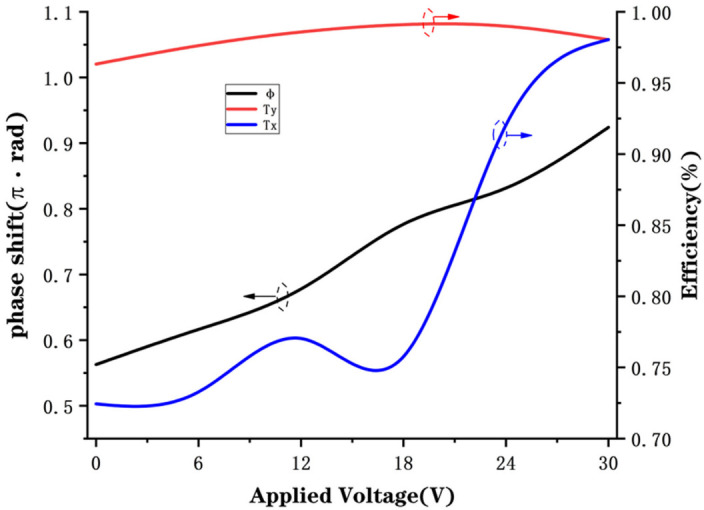
Simulated transmission of the metasurface and phase shift of emergent light as a function of applied voltage when the linearly polarized light normal incident to the metasurface. The black line represents the phase shift, the red line represents the transmission efficiency *T*x, and the blue line represents the transmission efficiency *T*y.

We performed simulations on the structures with the dimensions obtained above to obtain the phase shift and transmission by varying the refractive index of the BTO, which was calculated by Equation (1). Figure 4 shows the final simulation results with the optimized structural parameters (*H* = 590 nm, *D*x = 242 nm, *D*y = 100 nm, *P* = 350 nm), where the black line represents the phase shift ϕ, the blue line represents the transmission efficiency *T*x when a linearly polarized light occurred with polarization direction angle α=0, and the red line represents the transmission efficiency *T*y when incident a linearly polarized light with polarization direction angle α=π2, and the horizontal axis is the applied voltage, which is changed from 0 V to 30 V in 6 V steps for the purpose of eliminating the resonance point. The transmittance is the ratio of the power of the transmitted light to that of the incident light. It can be seen that when the applied voltage is not too large (0 ~ 30 V), the phase coverage reaches about π2, and the overall transmittance is higher than 72%.

**Figure 5:**
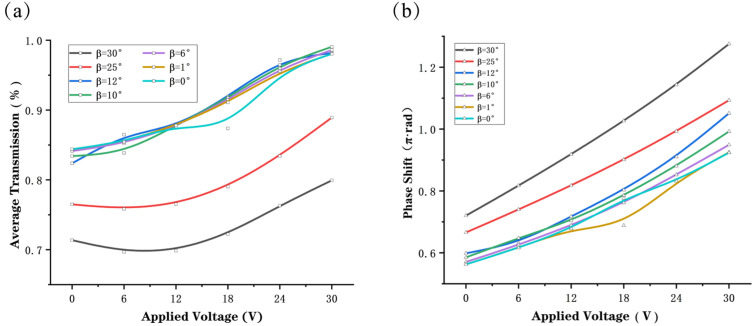
Simulated (**a**) average transmission and (**b**) phase shift of emergent light as a function of applied voltage when a linearly polarized light with polarization direction angle α=π4 incident to the metasurface at different angles of incidence β.

We also simulated what happens when light is incident obliquely, as shown in Figure 5. When the incidence angle is not equal to zero, the boundary conditions of the simulation need to be changed to: Block for the x-direction, Periodic for the y-direction, and PML for the z-direction.

From Figure 5a, we can analyze that, in general, the angle of the incident light β does not affect the coverage of the phase shift but as β increases, the phase shift deflects. Analysis of the data in Figure 5b show that the average transmission of metasurface decreases as the angle of incidence β increases from 0° to 30°. Although the incidence angle will decrease the efficiency of the device to some extent, the transmission rate of the metasurface is still greater than 70% as seen from the simulation data. This demonstrates that the metasurface depolarizer is insensitive to incident angle and can be widely used in practical applications.

In this paper, the metasurface structure acts as varies nanoscale waveplates with the phase shift ϕ which changes as the applied voltage changes. Under the established dimensions and condition, we can obtain a quarter-waveplate (ϕ=π2) when there is no applied voltage and a half-waveplate (ϕ=π) when applied a voltage of 30 V. It is known that at different voltages, the polarization states of the incident light will be scrambling in the domain of temporal passing through the metasurface depolarizer.

**Figure 6:**
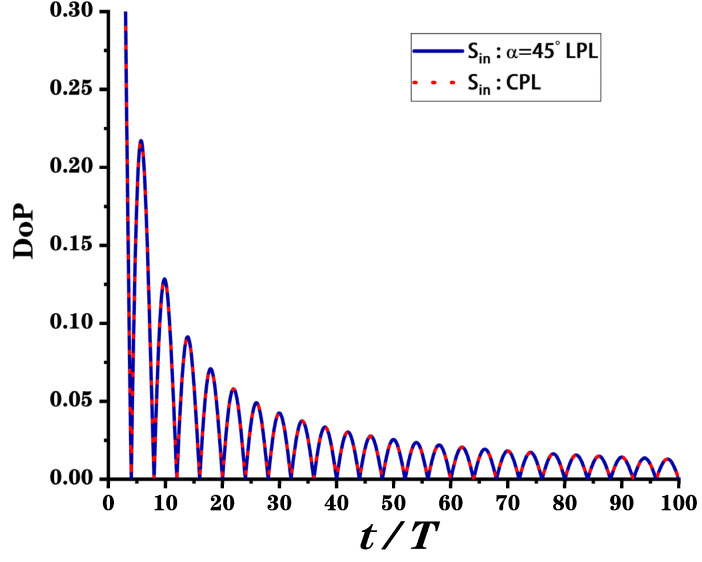
The DoP of ${\bar{S}}_{\mathrm{out}}$ with θ=0 and X=πt2T. The blue solid line is the result when the incident light is linearly polarized light with polarization direction angle α=π4; additionally, the red dotted line corresponds to the result when the incident light is circularly polarized light. The above two results are the same. LPL: linearly polarized light; CPL: circularly polarized light.

The depolarization performance of the metasurface element can be verified by calculations in the Mathematica (Wolfram Research, Champaign, IL, USA). We assume that the applied voltage varies from 0V to 30V according to the period T, in the other words, the phase shift of waveplate changes from π2 to π in the period of T and then X can be written as X=πt2T. Figure 6 shows the DoP of light emitted from the metasurface depolarizer after the time average by incident light of a linearly polarized light with α=π4 and circularly polarized light, respectively. The DoP corresponding to the two different incident lights is the same. Analysis of the data in the graph show that the depolarizer can be unpolarized light with a DoP = 5% starting from tT=25, and as time increases, the DoP will be further reduced to 2.5%. The effect of the depolarizer depends on the time *t* and the period T of the voltage change, which can be several microseconds with the development of modern electricity. Therefore, the response speed of this electronically controlled depolarizer can meet the development needs of ultrafast optics. Overall, the device we propose in this paper significantly reduces the DoP theoretically and will be a depolarizer with high performance and a compact structure.

## 4. Conclusions

In this paper, using the EO crystal BTO, we made an electronically controlled depolarizer based on the time-domain integral average method. This method achieves depolarization by ensemble averaging of multiple polarization states in the time domain, which keeps depolarizers free from the constraints of the light sources and device size. The electronically controlled method improves the methods from manual or mechanical ones to the electric automatic one; the latter makes the depolarizers more prominent and the operation more accurate and convenient. The use of BTO with an ultrafast-speed response and an increasingly fast slew rate in the electrical field makes this metasurface depolarizer stand out of the previous depolarizers scrambling polarization states in the time domain. Thanks to the advantages of ultra-thin and simple configuration, this kind of metasurface has the possibility to be applied to other systems in optics.

## Data Availability

Not applicable.
